# Sustainable Recycling of Selenium‐Based Optoelectronic Devices

**DOI:** 10.1002/advs.202400615

**Published:** 2024-03-15

**Authors:** Xia Wang, Zongbao Li, Bowen Jin, Wenbo Lu, Mingjie Feng, Binghai Dong, Qingxiang Liu, Hui‐Juan Yan, Shi‐Min Wang, Ding‐Jiang Xue

**Affiliations:** ^1^ Hubei Collaborative Innovation Center for Advanced Organic Chemical Materials Ministry of Education Key Laboratory for the Green Preparation and Application of Functional Materials School of Materials Science and Engineering Hubei University Wuhan 430062 China; ^2^ School of Materials Science and Engineering Wuhan Textile University Wuhan 430200 China; ^3^ School of Material and Chemical Engineering Tongren University Tongren 554300 China; ^4^ Beijing National Laboratory for Molecular Sciences (BNLMS) CAS Key Laboratory of Molecular Nanostructure and Nanotechnology Institute of Chemistry Chinese Academy of Sciences Beijing 100190 China; ^5^ University of Chinese Academy of Sciences Beijing 100049 China

**Keywords:** indoor photovoltaic, selenium, solar cells, thermal evaporation, thin film

## Abstract

Selenium (Se), the world's oldest optoelectronic material, has been widely applied in various optoelectronic devices such as commercial X‐ray flat‐panel detectors and photovoltaics. However, despite the rare and widely‐dispersed nature of Se element, a sustainable recycling of Se and other valuable materials from spent Se‐based devices has not been developed so far. Here a sustainable strategy is reported that makes use of the significantly higher vapor pressure of volatile Se compared to other functional layers to recycle all of them from end‐of‐life Se‐based devices through a closed‐space evaporation process, utilizing Se photovoltaic devices as a case study. This strategy results in high recycling yields of ≈ 98% for Se and 100% for other functional materials including valuable gold electrodes and glass/FTO/TiO_2_ substrates. The refabricated photovoltaic devices based on these recycled materials achieve an efficiency of 12.33% under 1000‐lux indoor illumination, comparable to devices fabricated using commercially sourced materials and surpassing the current indoor photovoltaic industry standard of amorphous silicon cells.

## Graphical abstract

A closed‐space evaporation process is reported to recycle Se and other functional materials from end‐of‐life Se‐based optoelectronic devices. This process achieves high recycling yields of ≈ 98% for Se and 100% for other functional materials including gold electrodes and glass/FTO/TiO_2_ substrates. The refabricated Se devices based on these recycled materials exhibit comparable performance to devices fabricated using fresh materials.

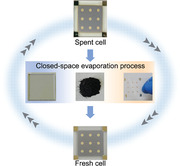

## 1 Introduction

Selenium (Se), the world's oldest optoelectronic material, has found extensive commercial use in various optoelectronic devices.^[^ ^1^, ^2^, ^3^, ^4^, ^5^ ^]^ Amorphous Se (a‐Se) stands as the currently market‐dominant photoconductive material in X‐ray imaging industry, particularly in X‐ray flat‐panel detectors for digital mammography.^[^ ^6^, ^7^ ^]^ Crystalline Se (c‐Se) is regaining significant attention as a promising absorber for top cells in multi‐junction solar cells and indoor photovoltaics (IPVs) due to its suitable wide bandgap of ≈ 1.9 eV,^[^ ^8^, ^9^, ^10^, ^11^, ^12^, ^13^ ^]^ excellent intrinsic phase and environmental stability to ambient conditions such as humidity, light and oxygen,^[^ ^14^, ^15^, ^16^ ^]^ and nontoxicity as an essential element for humans.^[^ ^17^, ^18^, ^19^ ^]^ In addition, the high piezoelectricity, thermoelectricity, and nonlinear optical responses of Se have also propelled its widespread use in various applications including wearable piezoelectric devices, thermoelectric devices, optical limiters, and field‐effect transistors.^[^ ^20^, ^21^, ^22^ ^]^

This expanding market for Se‐based devices thereby leads to an increased demand for Se materials. Unfortunately, Se is not an abundant element compared to its counterpart sulfur, with an abundance of about 0.05 ppm in the Earth's crust (Figure S1, Supporting Information).^[^ ^23^ ^]^ Even worse, Se is a dispersed element rarely found in its elemental form in nature, most occurring in sulfide ores.^[^ ^24^ ^]^ We therefore took the view that the abundant end‐of‐life Se‐based devices can serve as a source of high‐grade Se ores. Recycling Se as well as other valuable materials from spent Se‐based devices is thus quite necessary to achieve the sustainable development of Se‐based devices. However, current Se recycling methods predominantly focus on recovering Se from Se‐contaminated wastewater and selenides.^[^ ^25^, ^26^ ^]^ Recycling Se directly from element Se‐based devices has not been developed to date.

We reasoned that the principal obstacle in the recycling of Se from Se‐based devices is attributed to its low solubility in conventional solvents, a consequence of its pronounced covalent character.^[^ ^27^, ^28^ ^]^ Although this strong covalency imparts excellent environmental stability and absence of hysteresis in Se‐based optoelectronic devices, it results in limited solubility. We further ascertained that Se exhibits solubility in only a select few solvents, such as oleum, highly caustic solutions, and hydrazine.^[^ ^29^, ^30^ ^]^ However, the practical application of such highly acidic or alkalic solution and the inherently toxic and explosive nature of hydrazine significantly curtails their feasibility. The insolubility of Se thereby constrains the applicability of solution‐phase chemistry for Se recycling. This comparable challenge also exists in the recycling of decommissioned silicon solar panels arising from the robust covalent nature of silicon. Consequently, a sustainable recycling of Se from end‐of‐life devices is thus urgently required.

Here we introduce a closed‐space evaporation (CSE) strategy that utilizes the significantly higher vapor pressure of Se compared to other functional layers in Se‐based devices to recycle all of them from end‐of‐life Se devices. The very short distance between spent Se devices and top substrate (≈ 1 cm) prevents the loss of Se deposited on conventional large‐area vacuum chamber and enables the deposition of almost all Se vapors onto the top substrate, whereas other functional materials remain in evaporation source. By using this solvent‐free strategy, we achieve high recycling yields of ≈ 98% for Se and 100% for other functional materials including valuable gold electrodes and glass/FTO/TiO_2_ substrates. The refabricated photovoltaic devices based on these recycled materials exhibit an efficiency of 12.33% under indoor illumination at 1000 lux, comparable to devices fabricated using commercially sourced materials and superior to the current industry standard of commercialized amorphous silicon cells with indoor photovoltaic efficiency below 10%.

## 2 Results and Discussion

### 2.1 Recycling Strategy of Se from End‐of‐Life Se Devices

**Figure 1:**
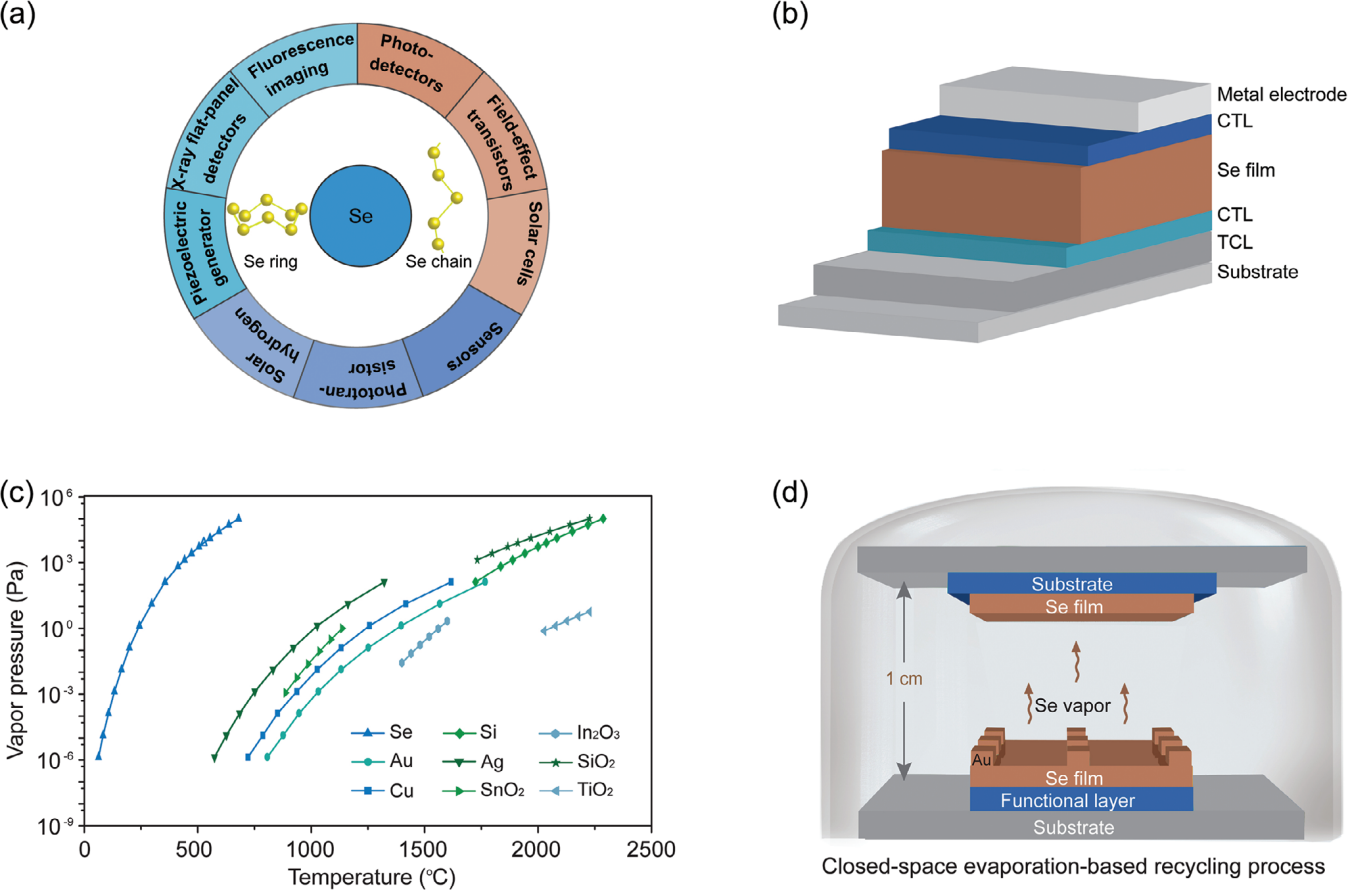
a) Summary of Se‐based optoelectronic and electronic devices. b) Schematic of Se thin‐film device architecture. c) Temperature‐dependent vapor pressure of commonly‐used functional materials in Se‐based optoelectronic devices. d) Schematic diagram of CSE process for recycling Se from Se‐based optoelectronic devices.

Se both in its amorphous and crystalline states have been widely applied in various thin‐film devices such as X‐ray flat‐panel detectors for digital mammography, top cells in multi‐junction solar cells, IPV cells for wireless Internet of Things terminal devices, wearable piezoelectric devices, field‐effect transistors, as well as other electronic and optoelectronic devices (**Figure** **1**a).^[^ ^2^, ^3^, ^4^, ^5^, ^6^, ^7^ ^]^ Most of these Se‐based thin‐film devices possess a conventional sandwich structure, wherein active Se layers are sandwiched in other functional layers such as charge transport layers (CTLs) including hole transport layers (HTLs) and electron transport layers (ETLs), transparent conducting layers (TCLs), and another metal electrodes (Figure 1b). When recovering active Se layers by conventional solution recovery processes using highly acidic or alkalic solution,^[^ ^27^, ^28^, ^29^, ^30^ ^]^ such solvents would disrupt the properties of other functional layers due to the potential chemical reactions between functional materials and solvents. From previously reported material cost analysis on Se‐based devices,^[^ ^31^ ^]^ we noted that other functional layers especially metal electrodes and CTLs also account for the dominant cost of whole Se devices. A physical strategy without involving chemical reactions that enables the efficient recovery of not only active Se layers but also all other valuable materials from end‐of‐life Se devices is thereby the most ideal choice.

We reasoned that the significant difference in vapor pressures between Se and other functional materials can provide an efficient physical method for recycling Se from spent Se‐based devices through vacuum distillation, where Se devices at the end‐of‐life can serve as evaporation source. Figure 1c compares the temperature‐dependent vapor pressure of Se and other functional materials.^[^ ^32^ ^]^ We observed that Se possesses significantly higher pressures at low temperatures (e.g., 100 Pa at 350 °C) compared to other commonly‐used functional materials in Se devices, such as a substrate (e.g., Si and SiO_2_), a transparent conducting layer (e.g., F‐doped SnO_2_ (FTO) or Sn‐doped In_2_O_3_ (ITO)), followed by a metal oxide charge transport layer (e.g., TiO_2_ and SnO_2_), and another metal electrode (e.g., Cu, Ag, and Au). This extremely high vapor pressure of Se at low temperature should be thereby suitable for recovering Se from Se devices via vacuum distillation, while this solvent‐free and low‐temperature process would avoid the potential risk of damaging other functional materials.

To effectively recycle Se and prevent the loss of Se deposited on conventional large‐area vacuum chamber, we designed a CSE process implemented in a traditional rapid thermal processing furnace; it is important to note that there exists an optimal distance (≈ 1 cm) between the end‐of‐life Se devices and the top substrate (Figure 1d). This optimal distance allows for the rapid deposition of almost all Se vapors onto the top substrate, thereby ensuring a high recycling efficiency of Se (Figures S2 and S3, Supporting Information). In this method, the end‐of‐life Se devices are used as the source for evaporation. Once heated, the Se active layer in devices would evaporate into a gaseous state, escape from the gap between adjacent functional layers (Figure 1b), and subsequently condenses onto the top substrate. In contrast, the other functional layers would remain in the source due to their low vapor pressure (Figure 1c). This process would thereby achieve the extraction of almost all Se from devices within one loop of recycling treatment while prevent the damage of other functional layers.

### 2.2 Recycling Process of End‐of‐Life Se Devices

**Figure 2:**
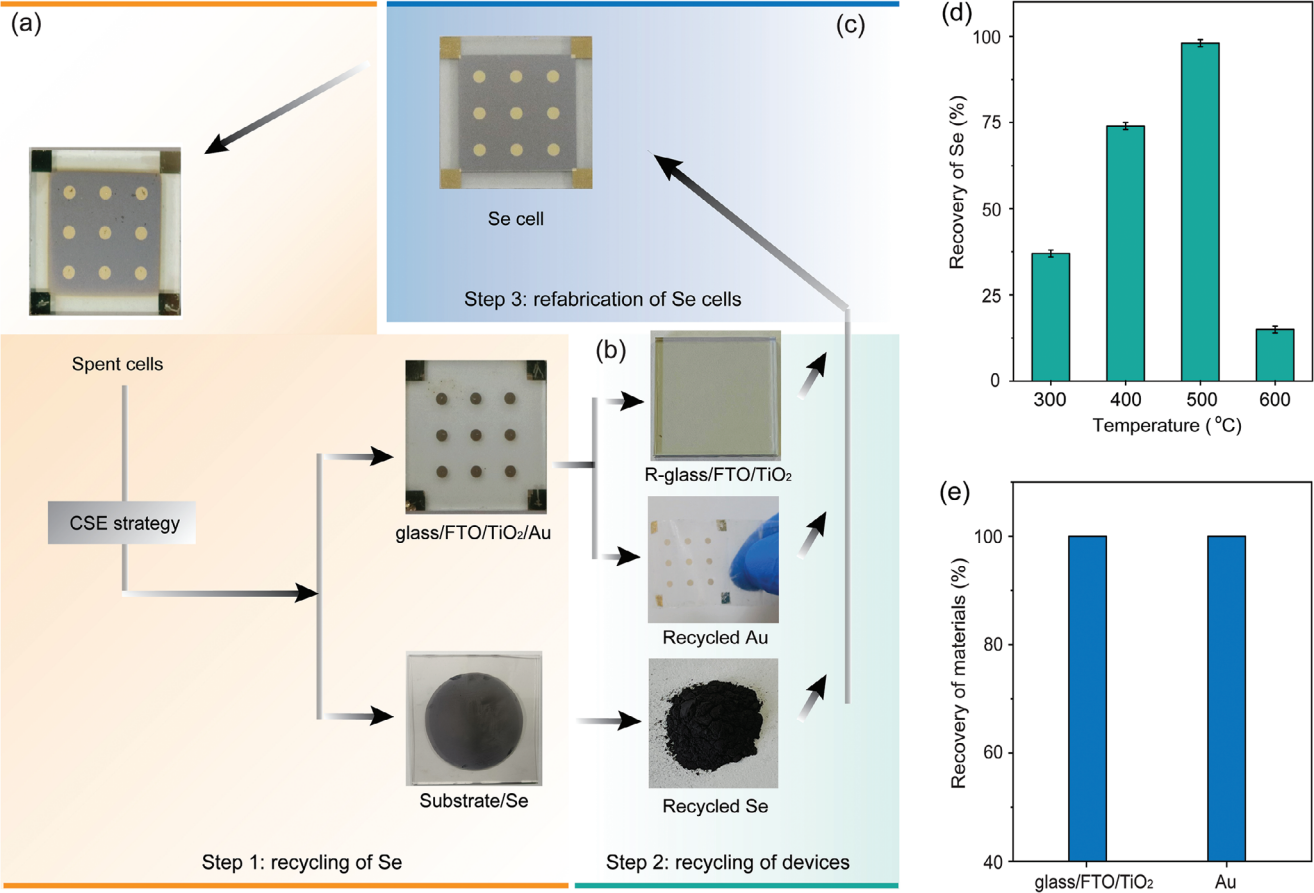
Roadmap for recycling whole Se solar cells: a) step 1: recycling of Se; b) step 2: recycling of devices; c) step 3: refabrication of Se cells. d) Recycling yield of Se via the CSE process at 300, 400, 500, and 600 °C, respectively. e) Recycling yield of glass/FTO/TiO_2_ substrate and Au.

Based on our proposed CSE process for recovering Se from Se devices, we further designed the recycling process of the whole Se optoelectronic devices, in the case of Se photovoltaic devices with a conventional structure of glass/FTO/TiO_2_/Se/Au. After the CSE process, the end‐of‐life Se film is removed from the TiO_2_‐coated glass/FTO (glass/FTO/TiO_2_) substrate and deposited on the adjacent top substrate (**Figure** **2**a). Se powder is ultimately obtained by scraping the Se‐contained substrate. Au electrodes are easily exfoliated from the glass/FTO/TiO_2_ substrate using ordinary tapes based on the removing of the sandwiched Se film, thus leaving the clean glass/FTO/TiO_2_ substrate (Figure 2b). The recycled Se, substrates, and electrodes would be subsequently reused in the fabrication of new Se optoelectronics with the same configuration (Figure 2c), thereby achieving the closed‐loop management for Se‐based devices.

We then optimized the evaporation temperature of CSE process that is a key parameter for this strategy. Based on the previously‐reported thermal gravimetric analysis of Se, the weight loss of Se initiates at ≈ 300 °C, and rapidly completes at ≈ 500 °C.^[^ ^33^ ^]^ The proper evaporation temperature might be thereby in the range from 300 to 500 °C. Figure 2d shows the recycling yield of Se at different evaporation temperatures (300, 400, 500, and 600 °C), where the recycling yield is calculated by the ratio of increased weight of top substrate to reduced weight of bottom end‐of‐life Se devices. 500 °C is observed to be the optimal evaporation temperature that leads to the highest Se recycling yield of ≈ 98%. In contrast, the recycling processes at 300 and 400 °C only lead to low recycling yields of about 37% and 74%, possibly arising from the low vapor pressure of Se at these temperatures. Despite the high vapor pressure of Se at 600 °C, the recycling yield at this temperature is only ≈ 15% mainly due to the re‐evaporation of condensed Se on top substrate at this high temperature. We thereby chose 500 °C as the source evaporation temperature in this CSE process. This temperature strikes a balance between the evaporation of Se at the source and the condensation of Se on the top substrate. After that, we further obtain 100% recycling yields of both Au and glass/FTO/TiO_2_ substrate (Figure 2e) arising from their significantly low vapor pressure at 500 °C, which are the primary cost components in Se solar cells. Furthermore, we found that this CSE process can be also extended to other end‐of‐life solar cells fabricated by high vapor pressure absorbers such as GeSe thin‐film solar cells (Figure S4, Supporting Information).

### 2.3 Characterization of Recycled Materials

**Figure 3:**
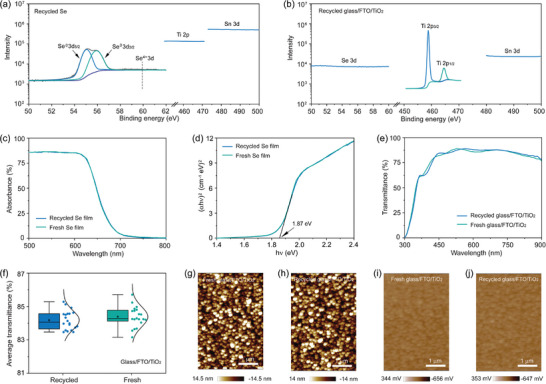
a) XPS spectra of Se 3d, Ti 2p and Sn 3d in the recycled Se. b) XPS spectra of Se 3d, Ti 2p, and Sn 3d in the recycled glass/FTO/TiO_2_ substrate. c) Absorption spectra of fresh and recycled Se films. d) Tauc plots of fresh and recycled Se films. e) Transmittance spectra of fresh and recycled glass/FTO/TiO_2_. f) Average transmittance of fresh and recycled glass/FTO/TiO_2_ in in the visible light range from 380 to 780 nm. AFM images of g) fresh and h) recycled glass/FTO/TiO_2_. KPFM potential difference images of i) fresh and j) recycled glass/FTO/TiO_2_.

To assess the quality of these recycled materials including Se and glass/FTO/TiO_2_ substrates, we employed X‐ray photoelectron spectroscopy (XPS) to examine potential impurities such as Ti and Sn elements in recovered Se powder and Se element in recycled glass/FTO/TiO_2_ substrates, respectively. Within the XPS detection limit of ≈ 0.1% atomic percent,^[^ ^34^ ^]^ no peaks corresponding to Ti and Sn, or Se are observed in the recycled Se powder or glass/FTO/TiO_2_ substrates (**Figure** **3**a,b), respectively. The results demonstrate the high purity of recovered Se, confirming the excellent effectiveness of our proposed CSE recycling process. We next investigated the suitability of these recycled Se for fabricating high‐quality Se films. Figure 3c compares the absorption spectra of fresh and refabricated Se films. It is clear that both fresh and recycled Se films display nearly identical absorption spectra, with a sharp decline starting at ≈ 630 nm and reaching almost zero at ≈ 700 nm. The two films show similar bandgaps of ≈1.87 eV (Figure 3d), agreeing well with previously reported values and indicating the undifferentiated optical properties of refabricated Se films.^[^ ^3^, ^31^ ^]^ The X‐ray diffraction (XRD) patterns and Raman spectra also confirm the desired photovoltaic state of trigonal phase for the refabricated Se films (Figure S5, Supporting Information),^[^ ^2^ ^]^ consistent with the reference Se film fabricated through fresh Se. The above undifferentiated material properties of recycled Se films and fresh Se films would thereby ensure the comparable performance of these refabricated Se devices to fresh devices.

We next carried out transmission spectroscopy to assess the recyclability of the recycled glass/FTO/TiO_2_ substrates. Figure 3e shows that no significant differences in transmission are observed between the recycled and fresh glass/FTO/TiO_2_ substrates, indicating the high quality of recycled glass/FTO/TiO_2_ substrates. We further calculated the average transmittance in the visible light range from 380 to 780 nm according to a previously reported formula.^[^ ^35^ ^]^ Figure 3f displays the statistical data collected from 20 substrates of each type, demonstrating the comparable average transmittance of recycled substrates to that of fresh substrates. We then evaluated the film quality of recycled TiO_2_ ETL, especially in terms of coverage, homogeneity, smoothness, and electrical properties, which are critical for optoelectronic device performance. We carried out both multi‐mode atomic force microscopy (AFM) and scanning electron microscopy (SEM) measurements on the recycled glass/FTO/TiO_2_ substrates. Figures 3g and h indicate that there are negligible differences in the surface morphology of the recycled and fresh glass/FTO/TiO_2_ substrates, consistent with the SEM images (Figure S6, Supporting Information). Figures 3i,j further compare the images of contact potential difference (CPD) between fresh and recycled glass/FTO/TiO_2_ substrates obtained from Kelvin probe force microscopy (KPFM). The nearly similar CPD values indicate that the recycled glass/FTO/TiO_2_ substrates possess similar band structure to that of the fresh substrates, with no additional trap states introduced during the CSE and exfoliating process. The above characterization thereby suggests the preserved quality of TiO_2_ layer on glass/FTO substrate with excellent coverage and homogeneity after recycling, consistent with fresh glass/FTO/TiO_2_ substrates, which can be directly used to refabricate new devices.

### 2.4 Performance of Refabricated Se Photovoltaic Devices

**Figure 4:**
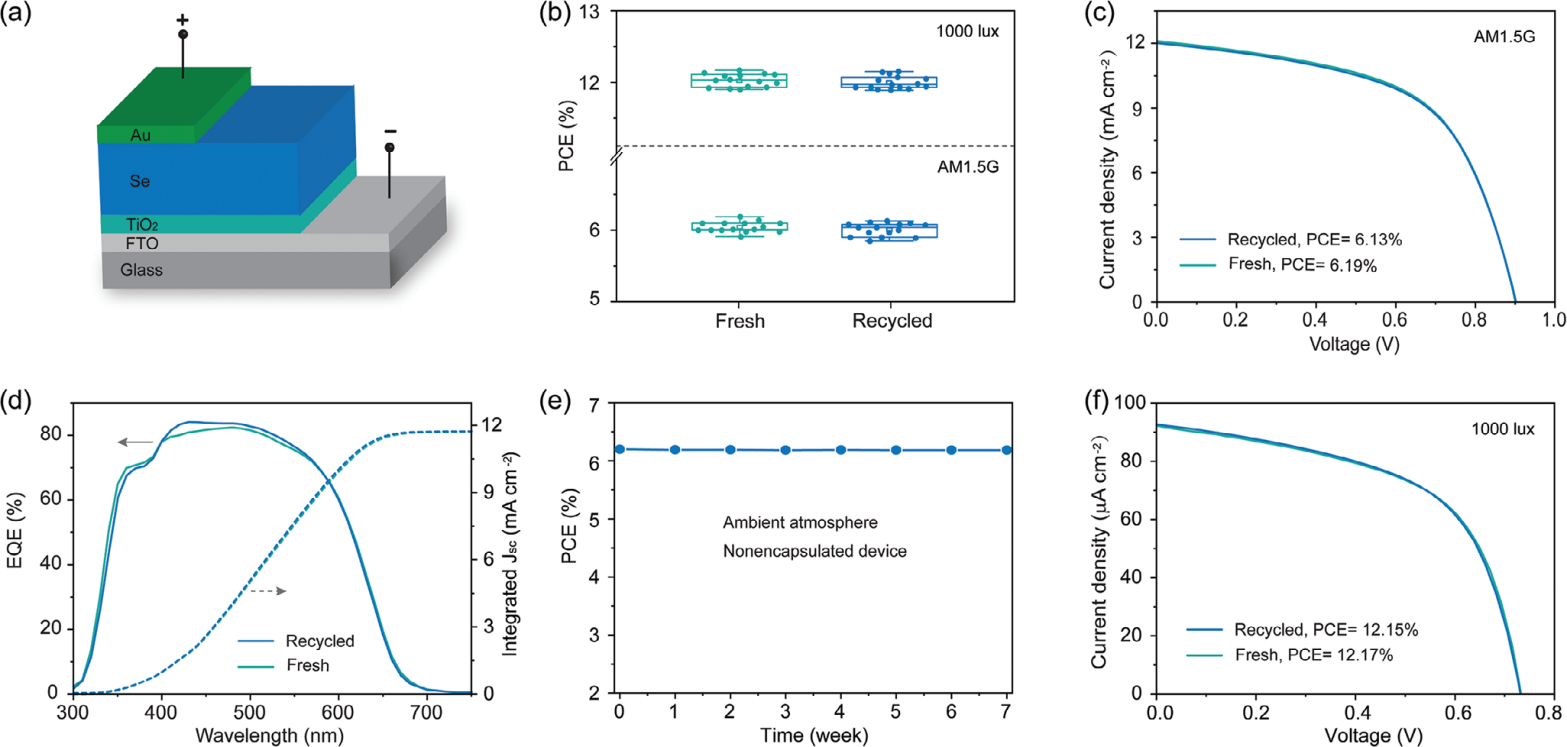
a) Schematic architecture of Se photovoltaics. b) PCE statistics of 15 Se photovoltaic devices fabricated using fresh and recycled materials, respectively. c) J–V curves of the best‐performing fresh and refabricated Se solar cells measured under standard AM1.5G illumination. d) EQE curve and integrated photocurrent density of the best‐performing fresh and refabricated Se solar cells. e) Evolution of PCEs of refabricated Se solar cell stored in ambient atmosphere. f) J–V curves of the best‐performing fresh and refabricated Se cells measured under indoor illumination from a LED at 1000 lux.

Such remarkable similarity between the fresh and recycled materials finally motivated us to utilize these recycled materials to refabricate conventional Se‐based solar cells structured as glass/FTO/TiO_2_/Se/Au (**Figure** **4**a). Figure 4b shows that such refabricated devices exhibit comparable performances to those devices fabricated using fresh Se and fresh substrates, demonstrating the excellent performance reproducibility associated with recycled materials. Figure 4c displays the current density‐voltage (J‐V) curves of the best‐performing fresh and refabricated devices under standard AM1.5G illumination at the intensity of 100 mW cm^−2^. The fresh and recovered Se devices exhibit comparable PCEs of 6.19% and 6.13%, respectively. The integrated photocurrent densities of the two types of Se devices obtained from the external quantum efficiency (EQE) spectra correspond well with the J_sc_ values measured from J‐V characterization (within 5% deviation) (Figure 4d). We further assessed the stability of the unencapsulated recycled Se devices stored in an air environment at room temperature with a relative humidity of 40% to 70%. It is observed that there is negligible PCE loss of the device after 7 weeks of storage (Figure 4e). This high stability of Se devices is benefited from the excellent stability of Se itself arising from its strong covalent character.^[^ ^33^ ^]^

We finally measured the IPV performance of such high‐efficiency refabricated and fresh Se devices using a commonly‐used warm white light‐emitting diode (LED) (2700 K), since the suitable wide bandgap (≈1.9 eV) and nontoxicity of Se make it ideal candidate for IPVs.^[^ ^3^, ^36^, ^37^, ^38^ ^]^ The corresponding input light intensity was measured to be 314.1 µW cm^−^^2^ from the emission power and integrated power spectra at 1000 lux (Figure S7, Supporting Information). All IPV measurements were performed in a homemade black box to prevent the interference of stray light, according to a reliable measurement method for IPVs reported by Hou group.^[^ ^39^ ^]^ Figure 4f shows the J–V curves of the best‐performing recycled and fresh Se devices. The recycled device exhibits a PCE of 12.15% with a corresponding V_oc_ of 0.73 V, J_sc_ of 92.47 µA cm^−2^, and FF of 56.63%. This IPV efficiency is comparable to that of the fresh device (12.17%), higher than that of current IPV industry standard of amorphous silicon (a‐Si) cells with indoor efficiencies below 10%.^[^ ^3^ ^]^ Overall, the nearly identical photovoltaic performances of the fresh and recycled devices confirm the effectiveness of our CSE strategy to recycle Se and other valuable materials from spent Se‐based devices, thus ensuring the sustainable development of Se optoelectronics.

## 3 Conclusion

In summary, we report a CSE strategy based on the highest vapor pressure of Se at low temperature among the functional layers in Se‐based devices. This strategy uses the end‐of‐life Se optoelectronic devices as evaporating source; the designed short distance between spent Se devices and top substrate (≈ 1 cm) enables the deposition of almost all Se vapors onto the top substrate while other functional layer materials remain in the source arising from low vapor pressure. This solvent‐free strategy achieves high recycling yields of ≈ 98% for Se and 100% for other functional materials including valuable gold electrodes and glass/FTO/TiO_2_ substrates. The refabricated photovoltaic devices based on these recycled materials exhibit an efficiency of 12.33% under 1000‐lux indoor illumination. This performance is comparable to the devices fabricated by commercial fresh materials, outperforming the present IPV industry standard of commercialized amorphous silicon cells with indoor photovoltaic efficiency below 10%.

## 4 Experimental Section

### Recycling of Se Devices

The Se‐recycling process was carried out by closed‐space evaporation in a rapid thermal processing furnace (MIT, Hefei, China). End‐of‐life Se optoelectronics were loaded on top of the AlN flake inside the quartz boat, and the glass substrate was suspended onto the quartz boat (≈ 1 cm above Se devices). Vacuum was maintained at 1 Pa using a simple mechanical pump. The recycling process was to quickly increase the source temperature from 25 to 300, 400, 500 or 600 °C within 20 s, respectively, maintain the predefined temperature for 30 s, then turn off the heating while introducing N_2_ into the tube furnace, and finally allow the sample to cool down naturally to room temperature. Note that a graphite lid was covered on the top of the top substrate. This lid was used to maintain the low temperature of top substrate during the rapid thermal processing of 20 s arising from the large heat capacity of graphite, thus avoiding the re‐evaporation of the Se films that had already deposited on top substrate.

### Solar Cell Fabrication

All devices were deposited on FTO conductive glass, which was ultrasonically washed using detergent, deionized water, acetone, and isopropanol for 40 min each. Substrates were cleaned by UV‐ozone treatment for 15 min. A compact TiO_2_ buffer layer was deposited onto the cleaned FTO class by hot‐air spray pyrolysis placing on a 450 °C hotplate, where a mixed solution of titanium diisopropoxide bis(acetylacetonate) (75% wt.% in isopropanol, Alladin) and absolute ethanol in the ratio of 1:9 by volume was used as precursor. This TiO_2_ film was then by annealing at 500 °C for 30 min and cooled down to room temperature naturally. Then, Se film was deposited on the FTO/TiO_2_ substrate by thermal evaporation under a vacuum pressure of 5 ×10^−4^ Pa. Au back‐contact electrodes (99.99%, Alfa Aesar, 10 nm min^−1^) with a thickness of 70 nm were deposited by thermal evaporation using a dot mask (0.09 cm^2^) under a vacuum pressure of 5 ×10^−4^ Pa. Finally, devices were completed by annealing on a preheated hot plate at 215 °C for 2 min under ambient conditions.

### Material Characterization

XPS measurements were performed on an ESCALab220i‐XL electron spectrometer (VG Scientific) using 300 W Al Kα radiation. Optical absorbance of Se film and transmission of substrates were measured using an ultraviolet‐visible‐near infrared spectrophotometer (UH4150, Hitachi). Powder XRD patterns were recorded using a Rigaku D/Max‐2500 diffractometer with a Cu target (Kα1 radiation, λ = 1.54056 Å). Raman spectroscopy (Horiba JobinYvon, LabRAM HR800) was measured under the excitation of 785 nm. AFM data was collected on a Bruker Dimension Icon microscope. Images of CPD were further performed using the KPFM mode in air at room temperature. SEM images were obtained by Hitachi S‐4800.

### Device Characterization

J‐V measurements of the devices were obtained using an AM1.5G solar simulator (100 mW cm^−2^, Newport, USA) equipped with a Keithley 2420 source meter and 450 W xenon lamp in air at room temperature. Light intensity was calibrated by a National Renewable Energy Laboratory certified Si solar cell with a KG−2 filter. The J–V curves were measured with a scanning rate of 100 mV s^−1^ (voltage step of 20 mV and delay time of 200 ms), recorded with both forward (−1 to 1 V) and backward (1 to ‐1 V) scans. The active area was determined by the aperture shade mask placed in front of the glass side of the solar cell with area of 0.03 cm^2^ to avoid overestimation of the photocurrent density. EQE spectra of the devices were measured by a QE‐R3011 PV measurement system (Enli Technology Co. Ltd). The emission spectrum, light intensity, and illumination of the indoor light source of warm white 2700 K LED were measured by a high‐precision fiber‐optic spectrometer (Maya‐2000Pro, Ocean Optics). The IPV measurement was performed in a homemade testing box in which all parts were painted black, while a baffle plate was designed to eliminate the effects from any stray lights.

## Conflict of Interest

The authors declare no conflict of interest.

## Supporting information

Supporting Information

## Data Availability

The data that support the findings of this study are available from the corresponding author upon reasonable request.
