# Supplementary material for: Sustainable Recycling of Selenium‐Based Optoelectronic Devices
Source: Adv Sci (Weinh). 2024 Mar 15;11(22):2400615. doi: 10.1002/advs.202400615 (PMC11165508; doi:10.1002/advs.202400615)
Supplement: Supplementary file 1 — Supporting Information [file ADVS-11-2400615-s001.pdf]

ADVANCED SCIENCE
Open Access

Supporting Information

for *Adv. Sci.*, DOI 10.1002/advs.202400615

Sustainable Recycling of Selenium-Based Optoelectronic Devices

Xia Wang, Zongbao Li, Bowen Jin, Wenbo Lu, Mingjie Feng, Binghai Dong, Qingxiang Liu, Hui-Juan Yan, Shi-Min Wang* and Ding-Jiang Xue**

Supporting Information

Sustainable Recycling of Selenium-Based Optoelectronic Devices

Xia Wang, Zongbao Li, Bowen Jin, Wenbo Lu, Mingjie Feng, Binghai Dong, Qingxiang Liu, Hui-Juan Yan, Shi-Min Wang,* and Ding-Jiang Xue**

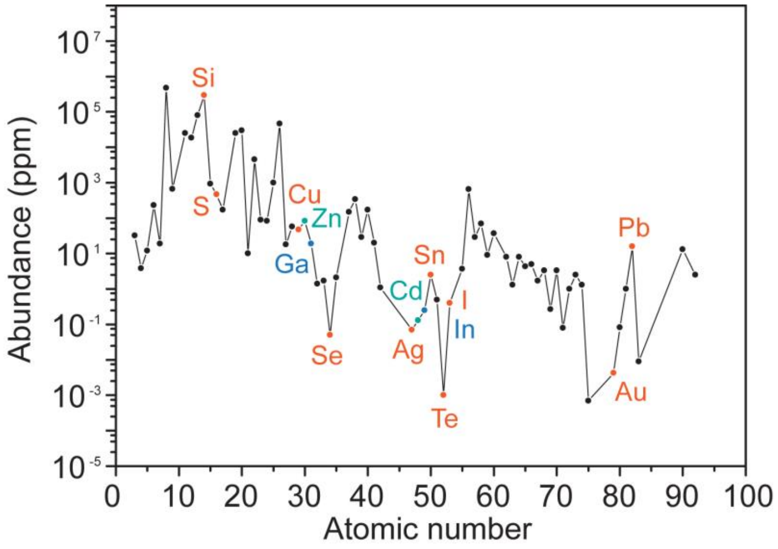

Figure S1. Summary of the elemental abundance.[1]

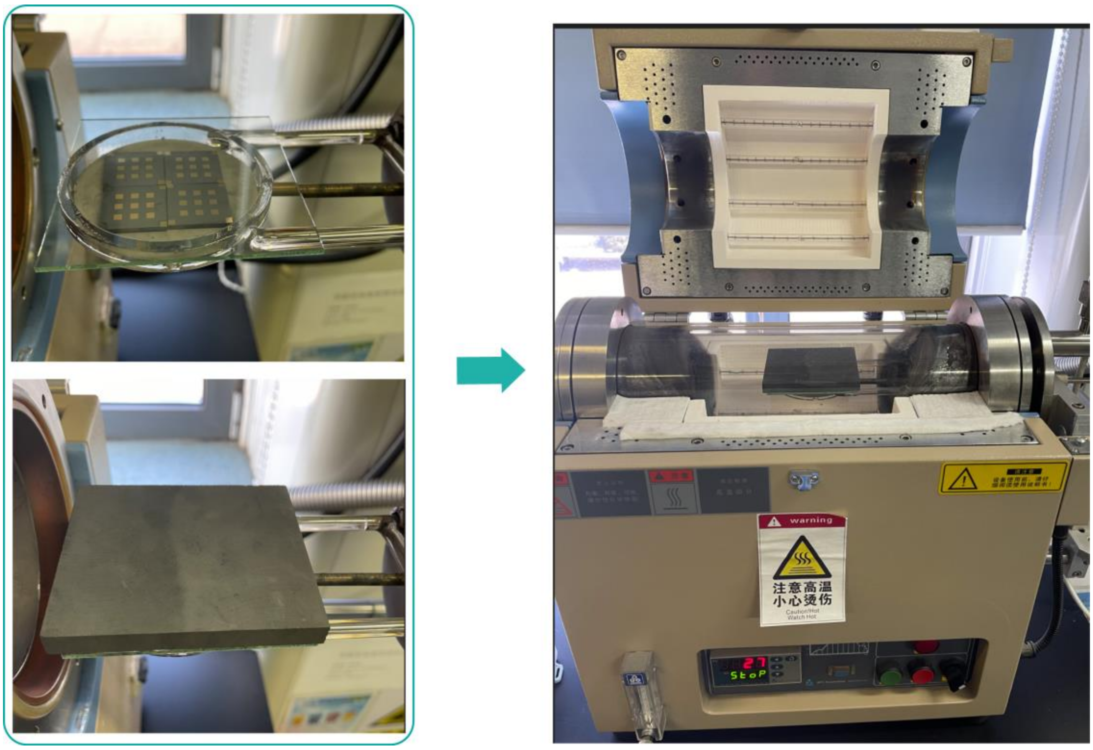

Figure S2. Photographs of the recycling Se system based on a rapid thermal processing furnace.

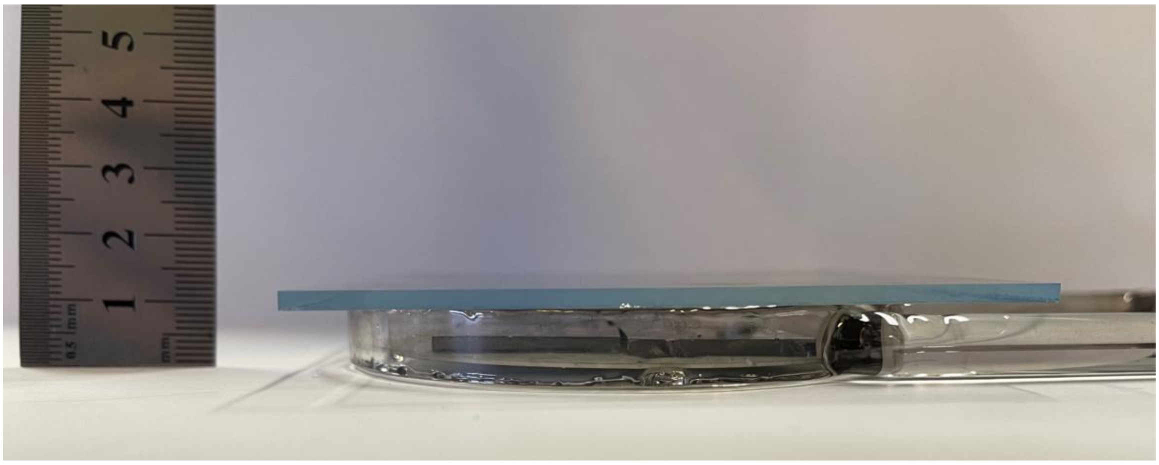

Figure S3. Photograph of our designed closed-space evaporation process.

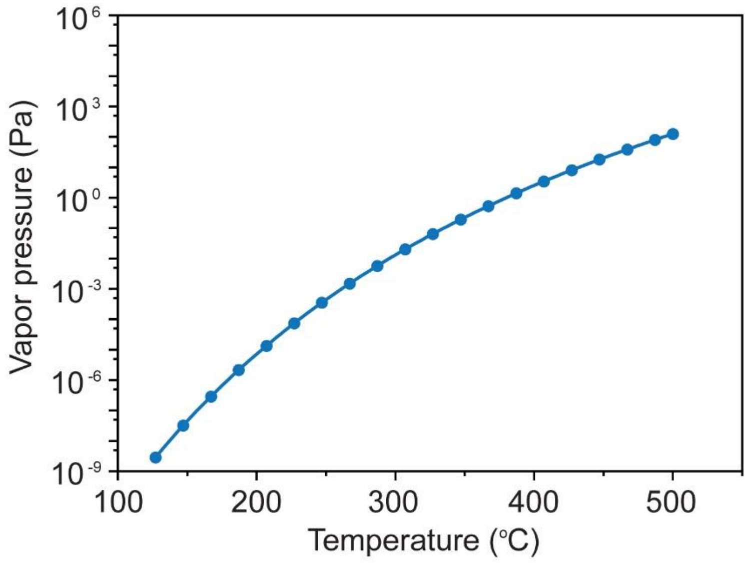

Figure S4. Temperature-dependent vapor pressure of GeSe.[2]

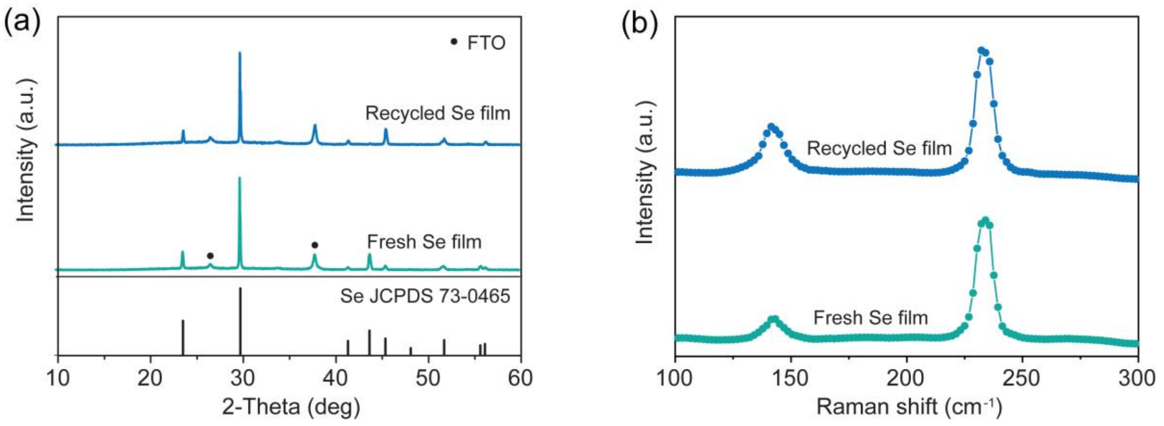

Figure S5. (a) XRD patterns of fresh and recycled Se films. (b) Raman spectra of fresh and recycled Se films.

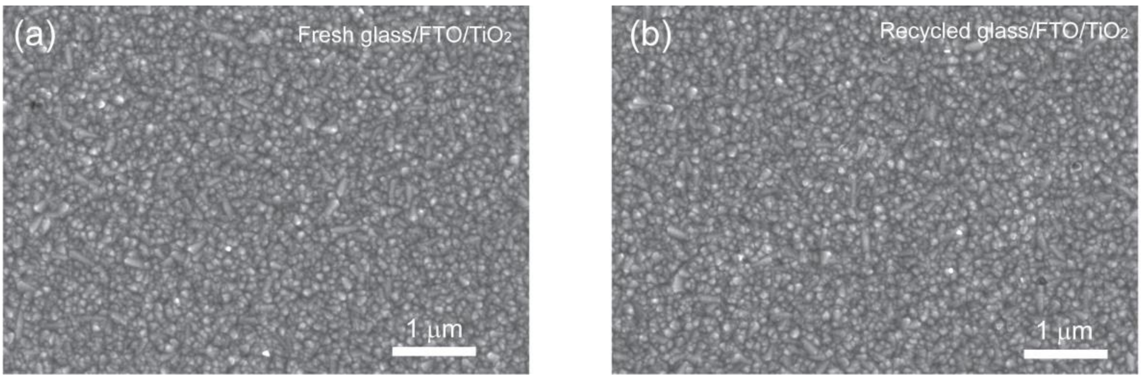

Figure S6. SEM images of fresh and recycled glass/FTO/TiO_2 substrates.

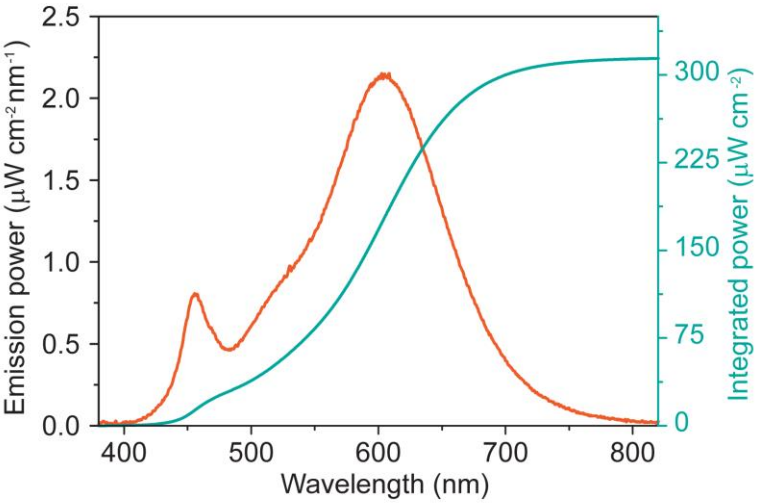

Figure S7. Emission power and integrated power spectra of a 2700 K LED at 1000 lux.

References in Supporting Information

[1] A. Yaroshevsky, *Geochem. Int.* **2006**, *44*, 48.

[2] D.-J. Xue, S.-C. Liu, C.-M. Dai, S. Chen, C. He, L. Zhao, J.-S. Hu, L.-J. Wan, *J. Am. Chem. Soc.* **2017**, *139*, 958.
